# The Cdk5 inhibitor Roscovitine increases LTP induction in corticostriatal synapses

**DOI:** 10.1042/AN20140006

**Published:** 2014-03-19

**Authors:** Jorge Miranda-Barrientos, Elizabeth Nieto-Mendoza, Elizabeth Hernández-Echeagaray

**Affiliations:** *Unidad de Biomedicina, FES-Iztacala, UNAM, México; †Programa de Doctorado en Ciencias Biomédicas, Universidad Nacional Autónoma de México, México

**Keywords:** Cdk5, dopamine, PKA, plasticity, Roscovitine, striatum, ACSF, artificial cerebrospinal fluid, AMPA, α-amino-3-hydroxy-5-methylisoxazole-4-propionic acid, Ca, calcium, Cdk5, cyclin-dependent kinase 5, DA, dopamine, DARPP-32, dopamine and cAMP-regulated phosphoprotein of 32 kDa, EPSC, excitatory postsynaptic currents, HFS, high-frequency stimulation, KA, kainate, LFS, low-frequency stimulation, LTD, long-term depression, LTP, long-term potentiation, MSN, medium spiny neurons, NMDA, **N**-methyl-D-aspartate, PKA, protein kinase A, PP1, protein phosphatase 1, PPR, pair pulse ratio, SP, substance P

## Abstract

In corticostriatal synapses, LTD (long-term depression) and LTP (long-term potentiation) are modulated by the activation of DA (dopamine) receptors, with LTD being the most common type of long-term plasticity induced using the standard stimulation protocols. In particular, activation of the D1 signaling pathway increases cAMP/PKA (protein kinase A) phosphorylation activity and promotes an increase in the amplitude of glutamatergic corticostriatal synapses. However, if the Cdk5 (cyclin-dependent kinase 5) phosphorylates the DARPP-32 (dopamine and cAMP-regulated phosphoprotein of 32 kDa) at Thr^75^, DARPP-32 becomes a strong inhibitor of PKA activity. Roscovitine is a potent Cdk5 inhibitor; it has been previously shown that acute application of Roscovitine increases striatal transmission via Cdk5/DARPP-32. Since DARPP-32 controls long-term plasticity in the striatum, we wondered whether switching off CdK5 activity with Roscovitine contributes to the induction of LTP in corticostriatal synapses. For this purpose, excitatory population spikes and whole cell EPSC (excitatory postsynaptic currents) were recorded in striatal slices from C57/BL6 mice. Experiments were carried out in the presence of Roscovitine (20 μM) in the recording bath. Roscovitine increased the amplitude of excitatory population spikes and the percentage of population spikes that exhibited LTP after HFS (high-frequency stimulation; 100Hz). Results obtained showed that the mechanisms responsible for LTP induction after Cdk5 inhibition involved the PKA pathway, DA and NMDA (N-methyl-D-aspartate) receptor activation, L-type calcium channels activation and the presynaptic modulation of neurotransmitter release.

## Abstract 2

Cdk5 inhibition favors striatal LTP induction through pre- and post-synaptic mechanisms.

## INTRODUCTION

Striatal MSN (medium spiny neurons) exhibit short-term and long-term plasticity (Surmeier et al., 2009). Synaptic plasticity can produce a reduction in the amplitude of synaptic response, called depression, or an increase in synaptic response amplitude, namely potentiation. It has been described that LFS (low-frequency stimulation) and HFS (high-frequency stimulation), respectively, produce striatal LTD (long-term depression), whereas LTP (long-term potentiation) is generated only with HFS or with some pharmacological manipulations (Calabresi et al., 1992a).

Striatal synaptic plasticity is modulated by DA (dopamine) (Calabresi et al., 1992b, 2000); the activation of D1 receptors increases cAMP and PKA (protein kinase A) levels. PKA, in turn, phosphorylates the DARPP-32 (dopamine-cAMP-regulated phosphoprotein of 32 kDa) at the Thr^34^ residue. When DARPP-32 is phosphorylated at Thr^34^, it inhibits the activity of protein phosphatase PP1 (protein phosphatase 1) (Hemmings et al., 1984; Nishi et al., 1999a). Nevertheless, if DARPP-32 is phosphorylated on its Thr^75^ residue, DARPP-32 becomes a potent inhibitor of PKA and D1 receptor signaling (Bibb et al., 1999).

It has been proposed that DARPP-32 controls the expression of striatal LTD and LTP (Calabresi et al., 2000). If the phosphorylation of DARPP-32 at its Thr^75^ residue inhibits PKA, we hypothesize that the kinase that phosphorylates the Thr^75^ residue in the DARPP-32 may participate directly or indirectly in the underlying mechanisms of striatal plasticity.

Cdk5 (cyclin-dependent kinase 5) phosphorylates DARPP-32 at the Thr^75^ residue (Bibb et al., 1999), and inhibits the D1 receptor signaling pathway. In the striatum, Cdk5 modulates synaptic transmission. Roscovitine, a potent Cdk5 inhibitor increases glutamatergic transmission in the striatum through the Cdk5/DARPP-32 pathway (Chergui et al., 2004); however, its role in synaptic plasticity has not yet been elucidated.

The present study demonstrated that Roscovitine increased the percentage of experiments that exhibit LTP induction, as well as the amplitude of population spikes recorded in glutamatergic corticostriatal synapses. Furthermore, LTP induced by Roscovitine is modulated by the activation of dopamine and NMDA (**N**-methyl-D-aspartate) receptors, PKA and L-type Ca (calcium) channels. It also demonstrated that plasticity induced by Roscovitine has a presynaptic and a postsynaptic component.

## MATERIALS AND METHODS

All experiments were carried out with the Institutional approval in accordance with the Mexican (NOM-062-ZOO-1999), Institutional (FES-I, UNAM, Ethics committee) and NHI regulations for the care and use of experimental animals (8^th^ Edition, Library of Congress, 2010940400).

### Animals

The mice used in this study were male C57BL/6 mice (Harlan, Inc., México) aged 4–5 weeks. They were housed in plastic cages, in groups of 5–6, in a room at 25°C with a 12/12 light-dark cycle, with free access to food and water.

### Electrophysiology

Electrophysiological experiments were performed on sagittal brain slices (300 μm) obtained as follow: animals were anesthetized (halothane) in an anesthesia chamber and were then decapitated. The brains were quickly removed and brain slices containing the dorsal striatum were cut using a vibrating microtome (1000 Plus Pelco vibratome; Ted Pella, INC.), in an immersed cold (4°C) low Ca^2+^ saline solution with the following composition (in mM): 130 NaCl, 2 KCl, 5 MgCl_2_, 1 CaCl_2_, 26 NaHCO_3_ and 10 glucose saturated with 95% (v/v) O_2_ and 5% (v/v) CO_2_ (298 mOsm/l, pH=7.4). After 1 h of recovery, slices used for field potential recordings were transferred to a submerge chamber and superfused with ACSF (artificial cerebrospinal fluid) at 34°C. The ACSF composition was (in mM): 125 NaCl, 26 NaHCO_3_, 1 MgCl_2_, 3 KCl, 2 CaCl_2_, 10 glucose, 0.2 Thiourea, and 0.20 ascorbic acid, gassed with 95% O_2_/5% CO_2_ (298–300 mOsm/l, pH=7.4).

Extracellular field recordings were performed in striatal cells by eliciting field excitatory postsynaptic synaptic spikes (population spikes) using a sharp (pencil-shaped), concentric, bipolar, tungsten-stimulating electrode (12 μm at the tip; FHC) attached to an isolation unit (DS2A, Digimiter LTD). The stimulating electrode was placed at the corpus callosum. The recording electrode was a borosilicate glass electrode (~5 MΩ resistance), containing NaCl (3M), and placed in the dorsal striatum, ~500 μm away from the stimulating electrode. A population spikes slope of 50% of the maximum was used for baseline. After 10 min of stable baseline, three trains of 3s, 100 Hz (tetanus) with intervals of 20 s were delivered. All experiments were recorded for at least for 30 min after HFS.

Slices used for whole cell recordings were transferred to a saline solution containing (in mM): 130 NaCl, 3 KCl, 2 MgCl_2_, 2 CaCl_2_, 26 NaHCO_3_, 1.2 NaH_2_PO_4_ and 10 glucose saturated with 95% O_2_ and 5% CO_2_ (298 mOsm/l; pH=7.4; 25–27°C). After 1 h of equilibration, single slices were shifted to a recording chamber that was continuously superfused with oxygenated saline (1–3 ml/min). Whole cell recordings were performed using an infrared differential interference contrast upright microscope (BX51WI, Olympus) coupled to a CCD (charge-coupled-device) camera. After field stimulation (as described above), EPSC (excitatory postsynaptic currents) were recorded in MSN through an Axopatch 200B amplifier (Axon, Molecular Devices), digitalized with the aid of a Digidata 1322A (Axon, Molecular Devices) and acquired through pClamp 9.1 software. Series resistance was compensated (80%). Input and access resistance were continuously monitored during the experiment by evoking a transmembrane current with a voltage command. Criteria for accepting recordings were an access resistance below 30 MΩ and no changes throughout all experiments. Patch micropipettes (3–6 MΩ) were pulled (Sutter Instruments, Inc.) from borosilicate glass tubes (1.5 mm OD, WPI) and were filled with internal saline containing (in mM): 72 KH_2_PO_4_, 36 KCl, 1.1 EGTA, 10 Hepes, 2CaCl_2_, 1 MgCl_2_, 2 ATP-Mg_2_, 0.3 GTP-Na and 5 QX-314 (272–275 mOsm/l; pH 7.3). All experiments were carried out in the presence of Bicuculline (10 μM) to block the GABAergic component. To evaluate the effects of Cdk5 inhibition, Roscovitine (20 μM) was added to the bath. Experiments designed to evaluate Cdk5 and signaling pathways were carried out in the presence of D1 and D2 receptor agonists and antagonists applied in the bath. To explore whether Cdk5 inhibition affects plasticity in both types of MSN D1-BAC mice were used, cells that did not exhibit fluorescence were assumed to be D2 expressing MSN. Finally, to identify if Cdk5 effects were mediated through an action on NMDA receptors or L-type Ca channels, some experiments were performed in the presence of AP5 (50 μM) or Nifedipine (5 μM), respectively. Data analyses were performed off-line with the aid of clampfit (Axon, Molecular Devices) and Microcal Origin (Origin Lab Corporation) software.

### Image acquisition

Fluorescent images were obtained using the upright microscope (BX51WI, Olympus) with a 20× objective magnification (Olympus X Lum PlanFl 20X/0.95W) coupled to a Hamamatsu (Orca C4742-95) camera, and acquired with the Olympus Image acquisition system Cell M®, which sent excitation light in the range 450–490 nm through a dichroic filter (495 nm); emission was 502–538 nm.

### Western blot

To evaluate the phosphorylation state of DARPP-32 in our experimental conditions, striatal slices were incubated with Roscovitine 20 μM or vehicle for 10 min in artificial brain fluid. After incubation, striatal tissue was homogenized in lysis buffer containing 26 mM Tris–HCl, 1% (v/v) Triton X-100, 1.3 M glycerol and 130 mM NaCl, with the protein phosphatase inhibitor cocktail Complete mini tab (Roche). The samples were recollected and centrifuged for 5 min at 4500 rpm; supernatants were recollected and store at −70°C. Protein quantification was made using the Bradford method and 80 μg of protein was loaded in 10% (w/v) PAGE for electrophoresis. Proteins were transferred to PVDF membranes and incubated with primary antibodies against pDARPP-32 Trh^34^ (1:1000), pDARPP-32 Trh^75^ (1:1000) and Actin (1:1000) for 12–20 h at 4°C, and with secondary antibodies (1:2000) with HRP for 2 h at room temperature. Protein detection was performed through the use of luminol with a chemiluminescence method (Millipore); images were acquired via a scan (HP Scanjet G2410) and analyzed with Image J software for the evaluation of densitometry.

### Reagents

All reagent were purchased from Sigma-Aldrich, except; antibodies, anti pDARPP-32 Trh^34^, anti pDARPP-32 Trh ^75^ (Millipore) and anti Actin-(Santa Cruz); and NaCl, KCl, NaH_2_PO_4_ from JT Backer.

### Statistical analysis

Data were analyzed with the statistical software Sigma Plot (Systat Software, Inc.) and plotted using Microcal Origin 7 (Microcal Origin Lab Corporation) and Adobe Illustrator 10 or Adobe® Creative Suite® 5 (Adobe Systems, Inc.). Statistical analysis was conducted with a parametric test or a non-parametric test if data did not display normal distribution. Data were considered statistically significant if *P*<0.05.

## RESULTS

### Plasticity of glutamatergic synapses

**Figure 1:**
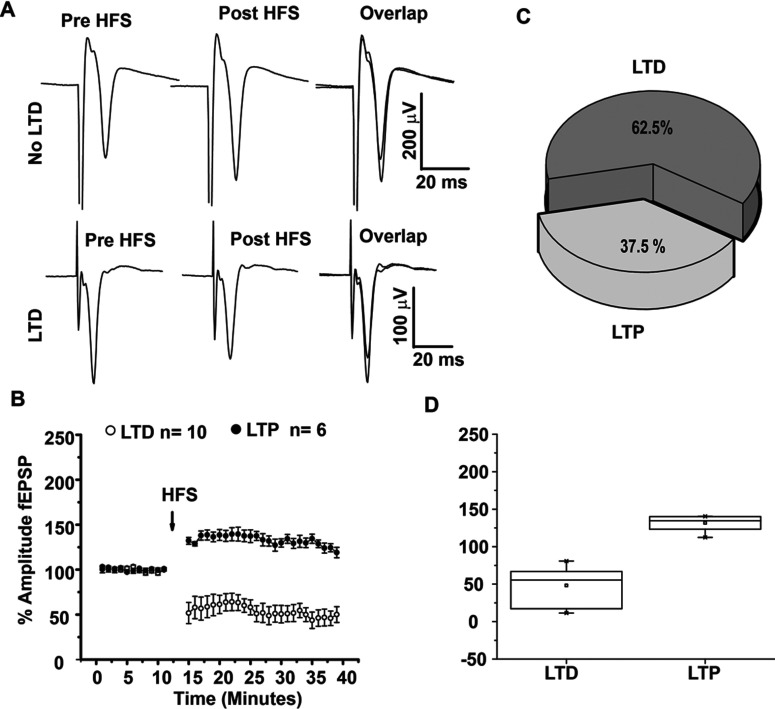
Plasticity of corticostriatal synapses (**A**) Population spikes traces show amplitude increase (LTP) or decrease (LTD) after HFS (three trains, 3 s, 100 Hz) of corticostriatal synapses. population spikes overlaps are magnified in the squares to illustrate population spikes before (black) and after (gray) HFS. (**B**) Temporal course of population spikes amplitude before and after HFS of corticostriatal synapses. Population spikes are normalized to 100% amplitude in control as a reference through all experimental conditions. (**C**) In control conditions, 62.5% of population spikes exhibited LTD and 37.5% displayed LTP. (**D**) Box plots illustrate the distribution of experiments where LTD or LTP was induced in comparison to control which was normalized to 100%. *P<0.0001.

HFS of the corticostriatal pathway produced both striatal LTD and LTP, as previously reported (Figures 1A and 1B), the percentage of experiments in which population spikes exhibited LTD after HFS was 62.5%, whereas LTP was observed in 37.5% of the recordings (Figure 1C). LTD observed in corticostriatal synapses was statistically significant (*t*_5_=6.458, *P*=0.000117, *t* test). The LTP observed in the 37.5% of the cases was also statistically significant compared to pre HFS (*t*_5_=−7.013; *P*=0.00094, *t* test, Figure 1D). Analysis of PPR (pair pulse ratio) of LTP or LTD was not statistically significant before and after HFS (*t*_8_=1.874, *P*=0.0977 and *t*_5_=2.22 *P*=0.0769, paired *t* test, LTD and LTP, respectively).

Comparisons of LTD with LTP obtained after HFS displayed statistical differences between (*t*_14_=7.402, *P*<0.0001, *t* test, Figure 1D).

### Cdk5 Inhibition produces an increase in glutamatergic population spikes and favors LTP induction after HFS protocol

**Figure 2:**
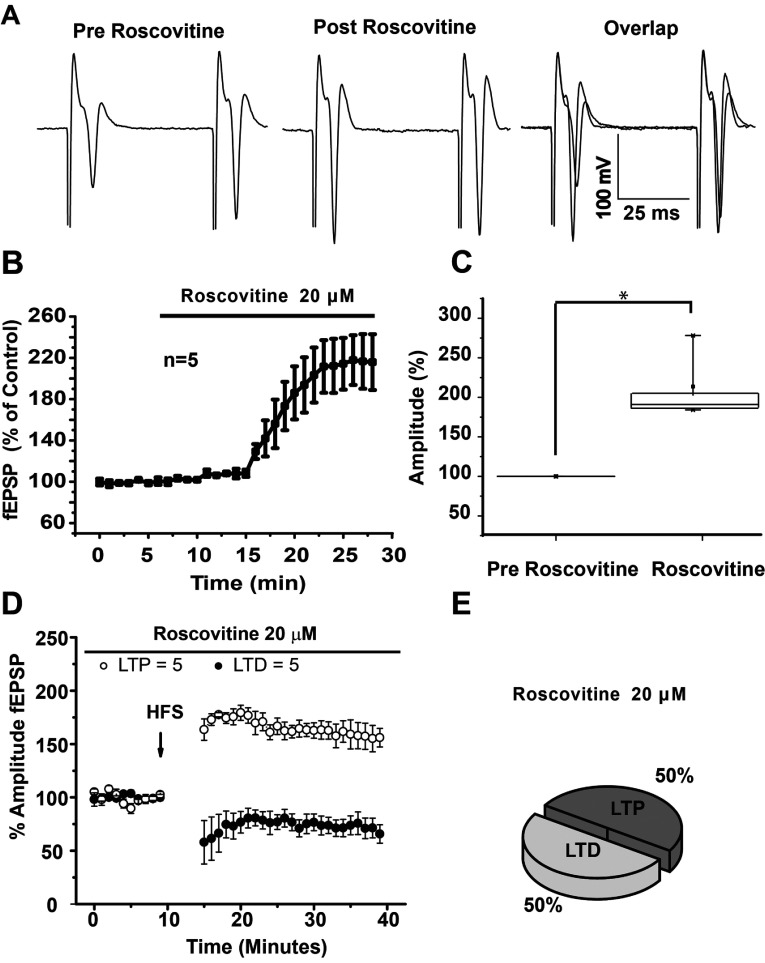
Roscovitine increases population spikes amplitude and facilitates the induction of LTP in glutamatergic corticostriatal synapses (**A**) Representative population spikes traces in control, in the presence of Roscovitine 20 μM, and the overlap of both conditions. (**B**) Temporal course of population spikes amplitude increase after bath application of Roscovitine (20 μM). (**C**) Box plot showing population spikes amplitude statistical changes in the presence of Roscovitine 20 μM. (**D**) Temporal course of field potential amplitudes before and after HFS. (**E**) Note that, in the presence of a Cdk5 inhibitor, there was an increase in the percentage of population spike that exhibited LTP and a reduction in the population spikes that exhibited LTD in the presence of 20 μM Roscovitine. *P<0.002.

We first sought to explore whether Roscovitine had a modulatory role in corticostriatal communication. Bath application of the Roscovitine (20 μM), significantly increased the amplitude of striatal glutamatergic population spikes in comparison to baseline (213.57±21.91 versus 99.99±0.005%, respectively; *t*_3_=−5.18; *P*=0.002, Figures 2A–2C). The analysis of PPR after bath application of Roscovitine (1.057±0.139), was significantly different (*t*_3_=10.300, *P*=0.00195, *t* test) to baseline (1.794±0.166), suggesting that Cdk5 inhibition modulates glutamatergic transmission through presynaptic mechanisms.

As the administration of Roscovitine alone generated an increase in the population spikes amplitude, we wondered whether Roscovitine might modulate the induction of striatal plasticity, then, HFS experiments were carried out in the presence of the Cdk5 inhibitor, Roscovitine. The percentage of cells that exhibit LTD and LTP with the HFS (100 Hz) protocol changed when Cdk5 was inhibited (Figures 2D and 2E), favoring LTP induction in 50% of experiments, while LTD induction was reduced 12.5% in comparison with control conditions. Then, in the presence of the Cdk5 inhibitor, 50% of the experiments displayed LTP and 50% of the experiments exhibited LTD. This suggests that Cdk5 activity in striatal MSNs is, in part, responsible for LTD generation after HFS.

### LTP induction in the presence of Roscovitine displays higher amplitude population spikes

**Figure 3:**
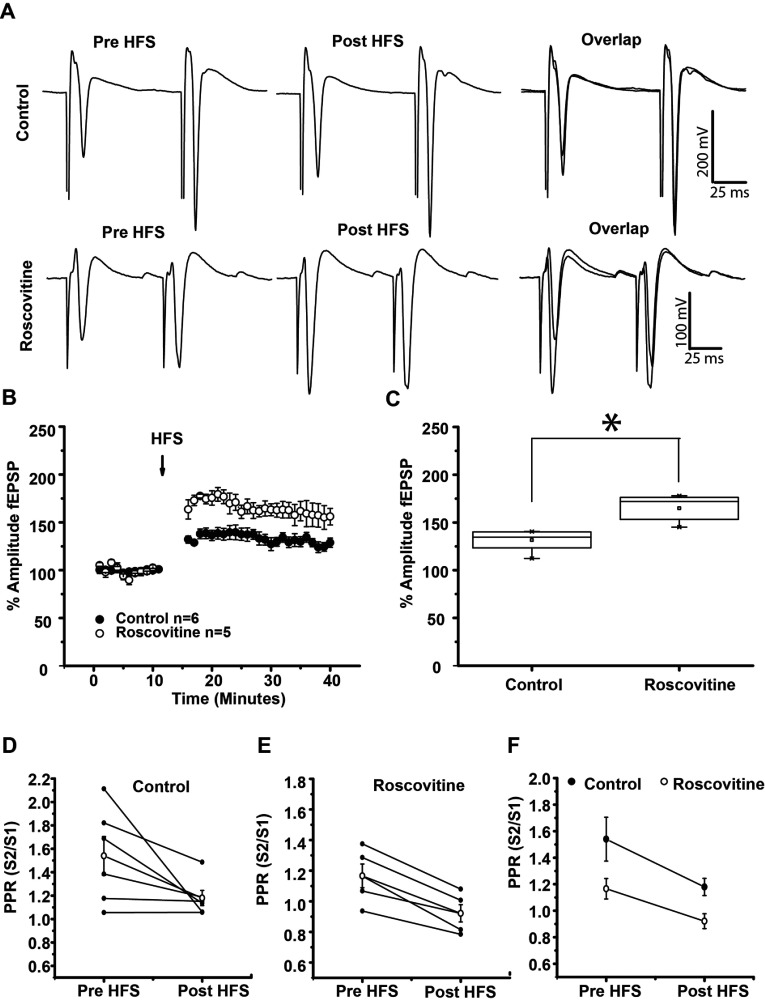
Population spikes amplitude increase in Roscovitine-induced LTP is greater than in control LTP (**A**) *Up*–Representative population spikes traces before HFS, after HFS and the overlap in control conditions (population spikes increased 31.544±4.643% after HFS). *Down*–Representative population spikes traces before HFS, after HFS and the overlap of both conditions in the presence of 20 μM Roscovitine. (Population spikes increased 64.864±6.528% after HFS in the presence of Roscovitine.) (**B**) Temporal course of population spikes amplitude increase before and after HFS (100 Hz, three trains, 3 s). Note the difference in LTP amplitude induced in control and that induced with Roscovitine (20 μM). (**C**) Box plot shows that population spikes amplitude increase in the presence of Roscovitine is statistically different from that of the control. (**D**–**F**) Illustrates PPR in control, in the presence of Roscovitine and the average of both experimental conditions. *P<0.03.

To further analyze LTP induction in the presence of Roscovitine, we compared LTP induced in control conditions with LTP induced in the presence of Roscovitine. population spikes increment produced by HFS in the presence of Roscovitine was 64.864±6.598% compared to baseline (figure 3B) these increment was statistically significant (*t*_4_=10.15 *P*=0.0005, paired *t* test). When we compare LTP in the presence of Roscovitine against LTP produced in control conditions (without Roscovitine), we found that LTP magnitude in presence of Roscovitine significantly increased in amplitude, in comparison to control conditions (Figures 3B and 3C) (LTP in control, 131.544±4.643% versus LTP in Roscovitine, 164.864±6.598%; t_9_=4.158, *P*=0.0025, *t* test, Figures 3B and 3C). The analysis of the PPR before versus after HFS in control conditions was not different (*t*_5_=2.222, *P*=0.077, *t* test, Figures 3D and 3F); on the contrary, PPR analysis in the presence of Roscovitine was statistically different (*t*_4_=6.010, *P*=0.00386, *t* test), suggesting that amplitude increase after HFS in the presence of Roscovitine may have a presynaptic origin (Figures 3E and 3F).

### LTP induction in the presence of Roscovitine is prevented in the presence of D1 and D2 antagonists

**Figure 4:**
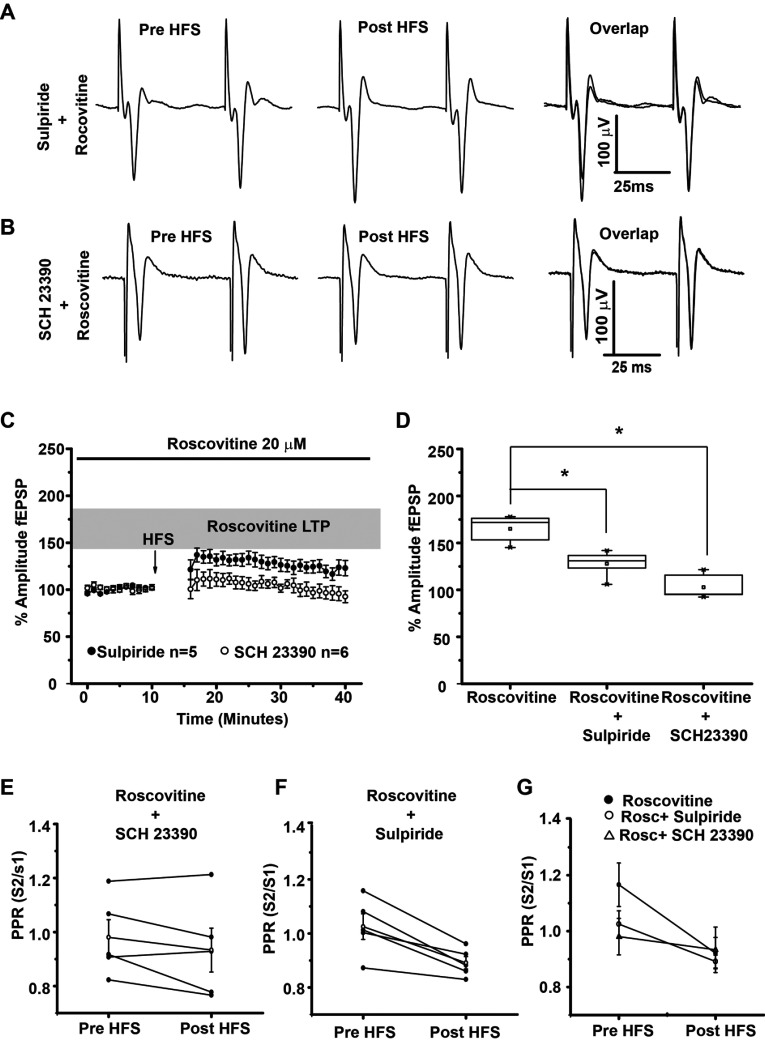
LTP induced with Roscovitine is prevented in the presence of D1 and D2 antagonists (**A**) Representative population spikes traces before and after HFS in the presence of 20 μM Roscovitine+1 μM Sulpiride. The overlap of traces shows that D2 antagonists prevented the amplitude increase in the population spikes triggered by HFS. (**B**) Representative population spikes traces before HFS and after HFS in the presence of 20 μM Roscovitine+1 μM SCH 23390. The overlap of traces shows that D1 antagonists prevented the amplitude increase in the population spikes triggered by HFS. (**C**) The temporal course of the population spikes amplitude increase after HFS, gray area shows LTP induced by Roscovitine (20 μM), open circles and closed circles shows population spikes in the presence of 20 μM Roscovitine+1 μM SCH 23390 and 20 μM Roscovitine+1 μM Sulpiride, respectively. (**D**) Box plot shows that the population spikes amplitude increase triggered by Roscovitine was prevented by D1 and D2 antagonists. (**E**–**G**) Illustrates PPR in the presence of 20 μM Roscovitine+1 μM SCH 23390, 20 μM Roscovitine+1 μM Sulpiride and the average of all experimental conditions. *P=0.004.

Previous experiments have shown that Cdk5 inhibits the PKA signaling pathway through DARPP-32 Thr^75^ phosphorylation (Bibb et al., 1999); therefore, since DARPP-32 plays an important role in DA signaling, we hypothesized that the underlying mechanism of LTP induction after Cdk5 inhibition might also involve DA receptor activation. As a result, we evaluated the role of D1 and D2 receptors in the population spikes increase with the co-application of specific antagonists for these receptors. Co-administration of Roscovitine (20 μM) and the D1 antagonist SCH 23390 (1 μM) blocked the LTP induction seen in the presence of Roscovitine alone (*t*_9_=7.562, *P*<0.0001, *t* test; Figures 4B–4D). In the presence of Roscovitine (20 μM) and the D2 antagonist Sulpiride (1 μM), the amplitude increase of population spikes induced with an HFS protocol was also significantly reduced [*t*_8_=4.076 *P*=0.0036, *t* test; Figures 4 (4C–4D)]. Analysis of PPR showed no statistical change between groups (*t*_4_=4.270, *P*=0.012 and *t*_4_=1.456, *P*=0.219, paired *t* test for sulpiride and SCH23390, respectively), suggesting a postsynaptic effect of DA receptor inhibition in Roscovitine LTP induction (Figures 4E–4G).

### The PKA inhibitor H-89 blocked LTP induction with Roscovitine

**Figure 5:**
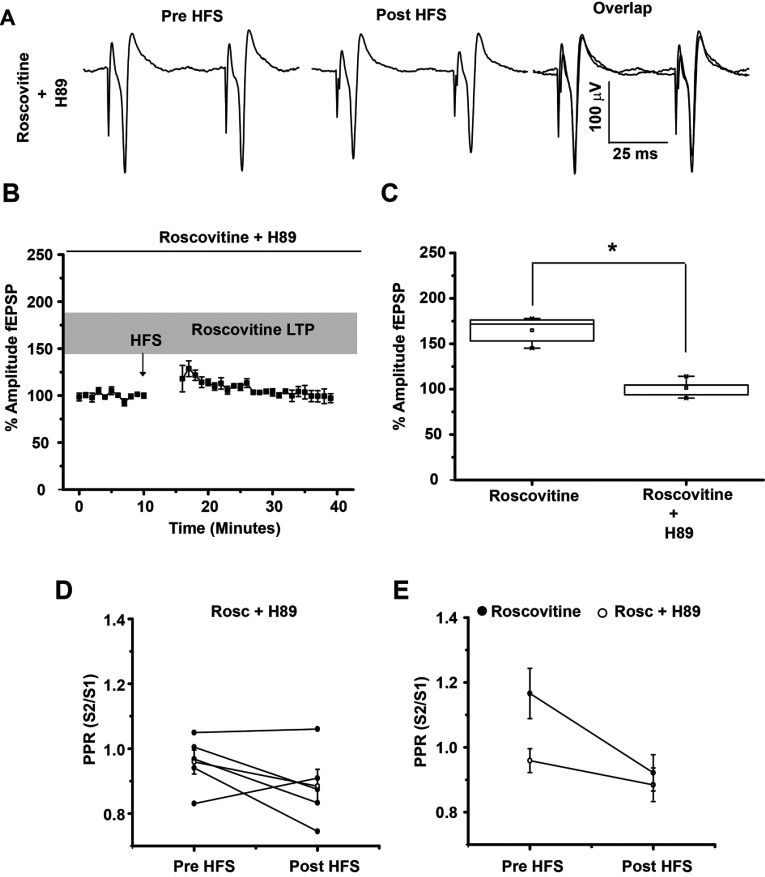
H-89 blocked LTP induction with Roscovitine (**A**) Representative population spikes traces before HFS and after HFS in the presence of Roscovitine (20 μM)+H 89 (5 μM). Population spikes traces overlap shows that H89 completely blocked any amplitude change. (**B**) Temporal course of population spikes amplitude increase after HFS (100 Hz) in the presence of 20 μM Roscovitine+5 μM H-89. (**C**) Box plot shows that population spikes amplitude increase triggered by Roscovitine was prevented by the PKA inhibitor H-89. (**D**–**E**) Illustrates PPR in the presence of Roscovitine+H-89 and the average of both experimental conditions (Roscovitine, Roscovitine+H-89). *P<0.03.

To evaluate whether PKA activation was necessary to induce LTP observed in the presence of Roscovitine, experiments were carried out in the presence of the PKA inhibitor H-89. Bath applied H-89 (5 μM) significantly prevented LTP induction in the presence of Roscovitine (*t*_8_=8.058, *P*=0.0001, *t* test, Figure 5). This data indicates that the PKA pathway is involved in LTP induction when Cdk5 is inhibited. The analysis of PPR before and after HFS in the presence H-89+Roscovitine suggested that this effect was postsynaptically mediated (*t*_4_=1.456; *P*=0.219, *t* test, Figures 5D and 5E).

**Figure 6:**
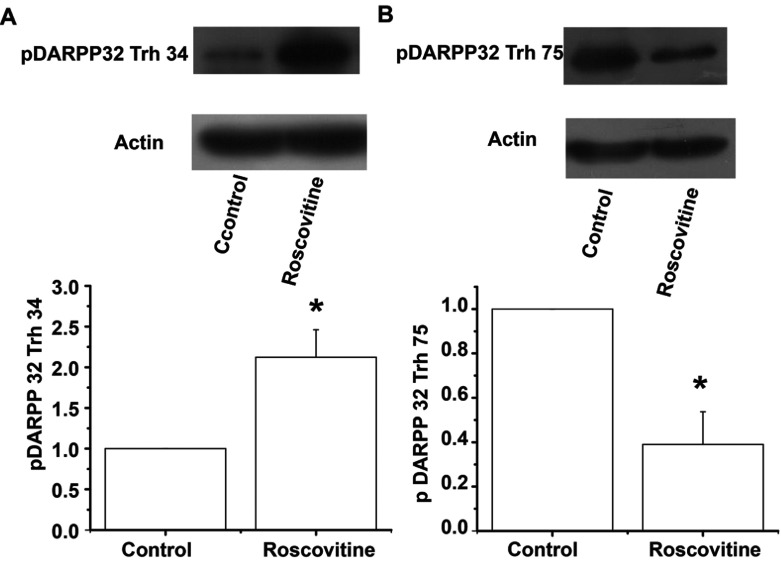
Western blots of pDARPP-32 obtained in striatal slices treated with Roscovitine The figure shows the bidirectional regulation of pDARPP-32 in the presence of Roscovitine. (**A**) In the presence of 20 μM Roscovitine, DARPP-32 Thr^34^ phosphorylation significantly increased compared to the control (2.12-fold compare to control). (**B**) In the presence of 20 μM Roscovitine, DARPP-32 Thr^75^ decreased 61% compared to the control. pDARPP-32 immunoblot levels were densitometrically scanned and represented as mean density/mean density of actin and expressed in arbitrary units (A.U.). Blots of pDARPP-32 Thr^34^ (1:1000), pDARPP-32 Trh^75^ (1:1000) and Actin (1:1000) are representative of at least three independent experiments of three mice brains each.*P<0.02.

H-89 is a potent PKA inhibitor, yet still exhibits a moderate inhibitory effect on other protein kinases (Meja et al., 2004); in order to better evaluate whether Cdk5 inhibition in slices modifies the phosphorylation state of DARPP-32, Western blot analysis was performed in striatal slices incubated with 20 μM Roscovitine. Phosphorylation of DARPP-32 at the residue Thr^34^ was greatly increased in the presence of Roscovitine (2.12-fold compare to control) (*t*_6_=3.337; *P*=0.0157, *t* test, Figure 6A), whereas DARPP-32 Thr^75^ exhibited a significant reduction of 61% (*t*_4_=4.137; *P*=0.0144, *t* test, Figure 6B) with the Cdk5 inhibitor, showing that DARPP-32 was less phosphorylated at Thr^75^ by Cdk5, leaving the PKA pathway active.

### NMDA and L-type Ca channel role in Roscovitine LTP induction

**Figure 7:**
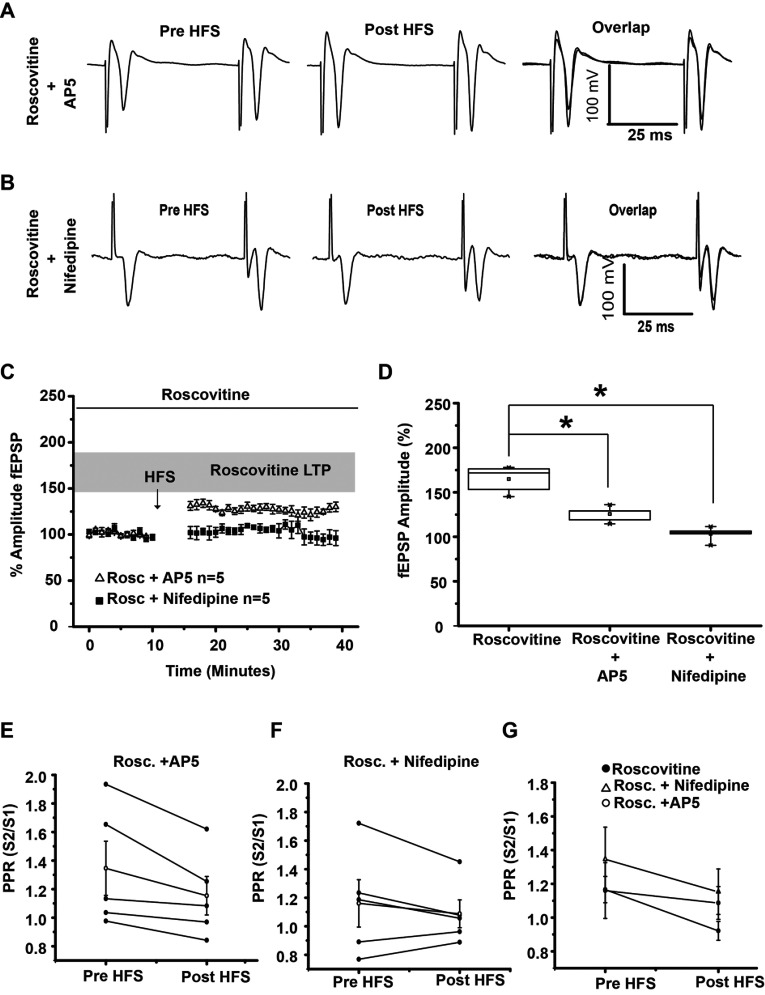
LTP induced with 20 μM Roscovitine is prevented in the presence of the NMDA receptor antagonist AP5 (50 μM) or L-type calcium blocker Nifedipine (10 μM) (**A**) Representative traces of population spikes in the presence of 50 μM AP5. AP5 reduced Roscovitine-LTP induction. (**B**) Representative traces of population spikes in the presence of 20 μM Roscovitine+10 μM Nifedipine. Nifedipine blocked Roscovitine-LTP induction. (**C**) Temporal course of population spikes in the presence of Roscovitine, Roscovitine+AP5 and Roscovitine+Nifedipine. (**D**) Box plot shows that population spikes amplitude increase triggered by Roscovitine was reduced significantly in the presence of AP5 or Nifedipine. (**E**–**G**) PPR in the presence of Rosco vitine+AP5, Roscovitine+Nifedipine is shown. (H) PPR analysis of the experimental conditions. *P<0.001.

Some of the cellular targets of PKA are NMDA and L-type Ca channels. To evaluate if Ca that enters through NMDA receptors and L-type Ca channels participates in Roscovitine LTP induction, NMDA receptors were blocked with AP5 (50 μM) in some experiments and L-type Ca channels were blocked with Nifedipine (10 μM) in other experiments; in both cases, population spikes was significantly reduced. AP5 significantly reduces LTP induced in the presence of Roscovitine (*t*_8_=5.142, *P*=0.0009, *t* test; Figures 7A, 7C and 7D) and Nifedipine abolished population spikes increase (*t*_8_=8.247 *P*<0.0001, *t* test; Figures 7B–7D). PPR analysis indicates that AP5 may have its effect at the presynaptic site (*t*_4_=2.76; *P*=0.05, *t* test, Figures 7E and 7G), whereas Nifedipine may have a postsynaptic effect (*t*_4_=1.456; *P*=0.219, *t* test, Figures 7F and 7G).

### Whole cell patch clamp recordings produced LTD in both D1- and D2-expressing cells

**Figure 8:**
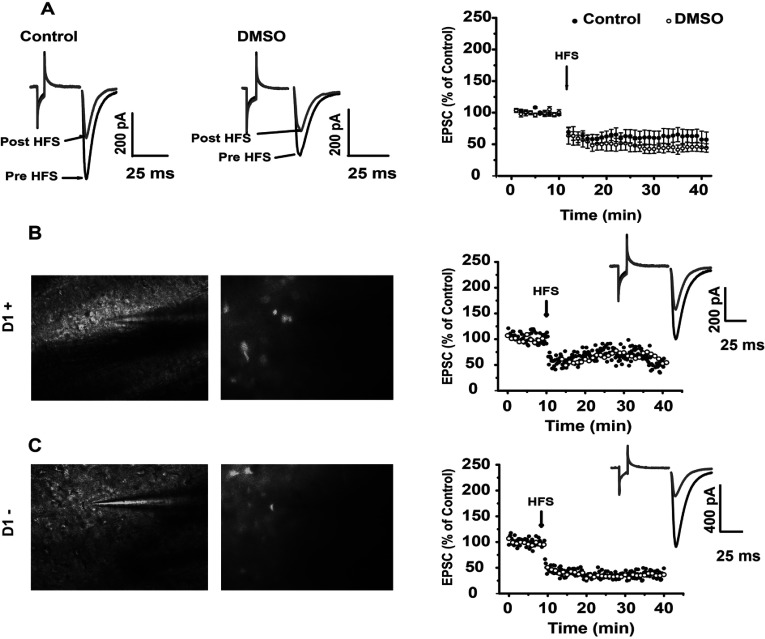
LTD was induced in D1+ and D1- medium spiny cells (**A**) DMSO did not affect MSN plasticity. (**B**) D1+ cells exhibited LTD after HFS. (**C**) D1-cells also exhibited LTD after HFS. In (**B**) and (**C**) the microphotography on the left was taken without GFP filter and with filter for GFP on the right photo. Inserts show LTD after HFS.

To evaluate whether all MSN are modulated by Cdk5, whole cell patch clamp recordings were carried out in D1-BAC mice. MSN that did not exhibit fluorescent emission were assumed to be D2-expressing neurons. Figure 8 illustrates that HFS produced LTD in most of the cells recorded, and the LTD produced was statistically significant (D1: *t*_4_=3.847; *P*=0.0184, *t* test; D2: *t*_5_=−2.649, *P*=0.0455, *t* test; Figures 8B and 8C, respectively). The vehicle used to dissolve Roscovitine did not affect this plasticity (Figure 8A).

### Switching off Cdk5 from the recording bath generates LTP in corticostriatal synapses of individual cells

**Figure 9:**
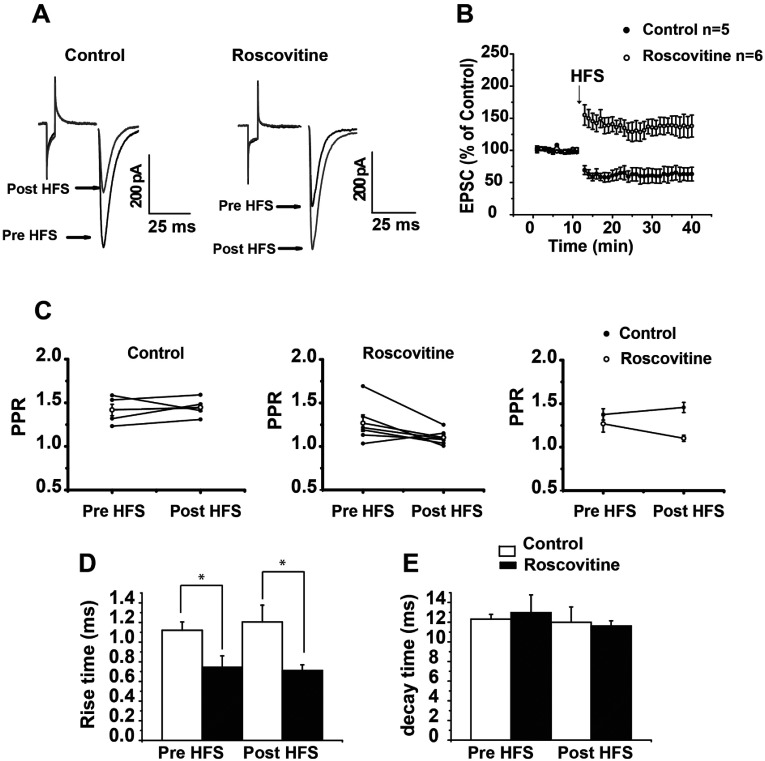
Switching off Cdk5 moves LTD to LTP in individual MSNs (**A**) Representative traces of EPSC in control and in the presence of 20 μM Roscovitine in the bath. The excitatory current decreased in amplitude after HFS, and increased in amplitude after HFS if Roscovitine was present in the recording bath. (**B**) Temporal course of EPSC after HFS in control conditions and in the presence of Roscovitine. (**C**) PPR did not change in control or in the presence of Roscovitine. (**D**) Illustrates the rise time of EPSC in the control compared to Cdk5 block. Rise time pre- and post-HFS significantly changed between groups. (**E**) Illustrates decay time EPSC in control compared to when Cdk5 was blocked. No changes were found between groups. *P<0.03.

When Roscovitine was added to the bath in whole cell recordings, HFS elicited only LTP (Pre-HFS 99.656±0.468%, versus post-HFS 138.107±12.421%; *t*_5_=3.066; *P*=0.0279, *t* test). Comparison of PPR before and after HFS was not significant in control conditions or in the presence of Roscovitine (Figure 9C), suggestive of postsynaptic changes after HFS. Time constant analysis of control or Roscovitine did not show changes before or after HFS; however, when the time constant of EPSC of the control was compared to EPSC in the presence of Roscovitine before (*t*_9_=2.544, *P*=0.0315, *t* test) or after HFS, it showed a significant difference (*t*_9_=2.945, *P*=0.0164, *t* test, Figure 9D).

Decay constants were not different between control pre- and post-HFS, nor between Roscovitine pre- and post-HFS or between groups both pre- and post-HFS (Figure 9E).

### Roscovitine facilitation of LTP has a presynaptic and postsynaptic component

**Figure 10:**
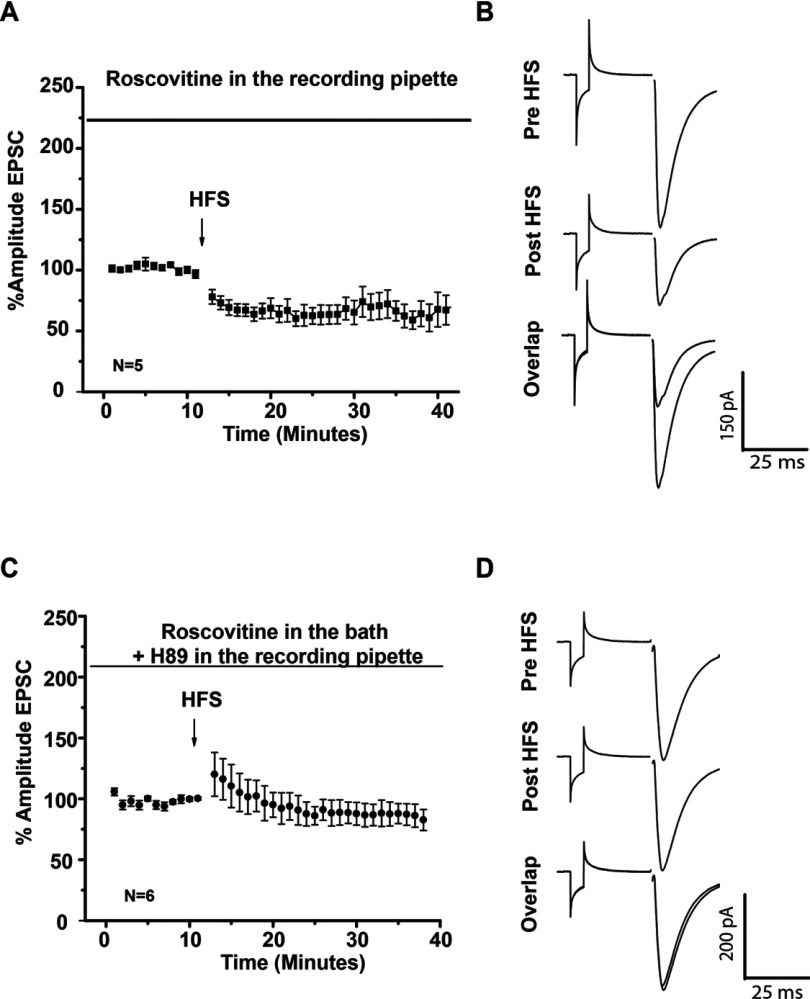
Presynaptic and postsynaptic component of striatal synaptic plasticity induced by de Cdk5 inhibitor Roscovitine (**A**) Temporal course of EPSC after HFS in the presence of Roscovitine in the recording pipette (10 μM). HFS protocol induced LTD. (**B**) Representative traces of EPSC in the presence of Roscovitine in the recording pipette. (**C**) Temporal curse of EPSC in the presence of Roscovitine (20 μM) in the recording bath and H89 (5 μM) in the recording pipette. Note that there were no changes in EPSC amplitude after HFS protocol. (**D**) Representative traces of EPSC in the presence of Roscovitine (20 μM) in the recording bath and H89 (5 μM) in the recording pipette.

Nonetheless PPR and time constant measurements in whole cell recordings suggested a postsynaptic component of Roscovitine LTP; It is known PPR is not a total convincing protocol to evaluate presynaptic effects when a postsynaptic component is involved. To test if there was a presynaptic and postsynaptic component in LTP induction after Cdk5 inhibition, two series of experiments were carried out. In the first series of experiments, whole cell recordings were made in the presence of Roscovitine in the recording pipette to inhibit Cdk5 activity in the postsynaptic cell. In this conditions, HFS induced LTD similar to control conditions (control 61.78±9.36% versus intracellular Roscovitine 67.06±6.92%, *t*_8_=0.4535 *P*=0.6622, *t* test), this data illustrated that Cdk5 has to be inhibited in the presynaptic site to induce LTP. In the second series of experiments, Roscovitine was added in the recording bath to inhibit Cdk5 at the presynaptic site and the PKA inhibitor H89 was in the recording pipette to inhibit PKA pathway in the postsynaptic cell. In this conditions, HFS did not induce long-term plasticity (pre-HFS=98.18412±1.192 versus post-HFS=84.247±8.01, *t*_5_=1.628 *P*=0.1644, *t* test), showing that the PKA signaling pathway is associated with to the postsynaptic component of Roscovitine-induced LTP (Figure 10).

## DISCUSSION

The molecular process underlying synaptic plasticity in the striatum has not been fully elucidated; in this paper, we evaluated the modulatory role of Cdk5 on corticostriatal synaptic plasticity. Our results show that the inhibition of Cdk5 with the inhibitor Roscovitine favors the striatal excitability of population spikes and facilitates LTP induction of glutamatergic transmission.

### HFS of corticostriatal synapses, produced both LTD and LTP

LTD is the most frequent type of striatal plasticity however, as previously reported we obtained LTD mostly, but also LTP with the same stimulation protocol. It has been shown that back propagate action potentials into the higher-order dendrites of striatal neurons during cortically driven ‘up’, together with spike-timing relative to cortical excitatory inputs can induce and determine the direction of synaptic plasticity at corticostriatal synapses (Pawlak and Kerr, 2008). Then depending on the activity history together with intracellular signals of a neuron, one form of plasticity or another will be induced. This study evaluated how striatal plasticity can be modified by Cdk5 signaling pathway.

### Cdk5 modulates glutamate release in corticostriatal synapses

Cdk5 inhibition with Roscovitine increased the amplitude of the excitatory population spike. It was previously reported that Cdk5 inhibition increases NMDA-activated currents (Chergui et al., 2004); however in our experimental conditions, population spikes were recorded and mediated mainly by AMPA (α-amino-3-hydroxy-5-methylisoxazole-4-propionic acid)/KA (kainate) activation, in fact Mg^2+^ was always present in the extracellular media. The reduction in the spike PPR in the presence of Roscovitine indicated that, the amplitude increase of the population spike had a presynaptic component. Cdk5 activity reduces glutamate release through different mechanism. For example, the inhibition of Cdk5 increases neurotransmitter release by modulating fusion pore (Barclay et al., 2004), mobilizing the storage pool (Kim and Ryan, 2010) or by modulating N and P/Q Ca channels (Tomizawa et al., 2002; Su et al., 2012). In the striatum, glutamate release from corticostriatal terminals is controlled by N and P/Q Ca channels (Bargas et al., 1998); and, these channels could be a phosphorylation target of Cdk5 in corticostriatal terminals.

### Cdk5 activity is involved in striatal plasticity

Our results show that Cdk5 activity is important for LTD induction because Cdk5 inhibition turned LTD into LTP. What is more, the corticostriatal LTP produced with HFS when Cdk5 is inhibited with Roscovitine was greater in magnitude than the corticostriatal LTP that is occasionally obtained in control slices. Cdk5 is important for the induction of LTD (Ohshima et al., 2005) and LTP (Li et al., 2001) in the hippocampus; furthermore, in conditional Cdk5 mice there is an LTP increase (Hawasli et al., 2007); however, there were any reports that demonstrated that Cdk5 inhibition favored striatal LTP and its activity participated in corticostriatal LTD.

### LTP induced by Cdk5 inhibition is modulated by dopamine

Owing to Cdk5 inhibition favored striatal LTP induction, signaling pathways modulated by Cdk5 should participate in corticostriatal plasticity. DARPP-32 is target of Cdk5 as well as PKA, and activation of the PKA signaling pathway increases NMDA currents (Flores-Hernández et al., 2002) then, it is possible that PKA activation favored striatal LTP, while the activation of Cdk5 maintained LTD. Our results indicated that striatal LTP induced by the inhibition of Cdk5 with Roscovitine involved the D1 signaling pathway because, in the presence of the D1 antagonist SCH23390 or the PKA inhibitor H-89, Roscovitine LTP induction was no longer generated in corticostriatal synapses even using the HFS protocol. Interestingly, the blockage of LTP by D1 inhibitors did not re-establish LTD, which suggested that Cdk5 may modulate LTD by means of an independent mechanism from that which LTP is modulated.

Besides D1 involvement in striatal LTP, we found that the D2 receptor antagonist Sulpiride diminished the magnitude of LTP induced by Roscovitine. D2 activation has been related to the generation of striatal LTD (Centonze et al., 2001; Lovinger, 2010); nevertheless, the D2 signaling pathway can stimulate PKC in medium spiny cells (Surmeier et al., 2007), which has been associated with striatal LTP (Gubellini et al., 2004). Moreover, D2 activation results in changes to the phosphorylation state of both Thr^75^ and Thr^34^ residues of DARPP-32 (Nishi et al., 1999b), which are the targets of Cdk5 and PKA, respectively. However, we are not ruling out that other protein kinases or phosphatases participate in LTP induction by Cdk5 inhibition. For example, D2 stimulation activates the PP-2B (protein phosphatase 2B), also known as calcineurin, which in turn modulates PKA/DARPP-32 pathways (Greengard et al., 1999). Therefore an indirect action of D2 on PKA signaling cannot be ignored in corticostriatal LTP.

### Cdk5 modulates striatal plasticity through the phosphorylation of Trh^75^ residue of DARPP-32

Phosphorylation of DARPP-32 at the Thr^75^ residue (Bibb et al., 1999; Chergui et al., 2004), converts DARPP-32 into a potent inhibitor of PKA (Bibb et al., 1999); this action stops the cellular effects produced by D1 receptor activation. However, if Cdk5 is inhibited, the D1 signaling pathway should prolong PKA activity. We tested this idea by analyzing pDARPP-32 Thr^34^ and pDARPP-32 Thr^75^. In the presence of Roscovitine, Thr^34^ residue increased while Thr^75^ residue reduced, providing evidence that the inhibition of Cdk5 favors the phosphorylation state of DARPP-32 Thr^34^ which may be responsible for LTP induction in the present report. This information is also supported by data obtained from experiments where D1 receptor and PKA activity were blocked in the presence of Roscovitine and LTP induction was prevented in corticostriatal synapses.

### L-type channels and NMDA receptors are involved in Roscovitine LTP induction

The inhibition of L-type Ca channels or NMDA receptors with Nifedipine and AP5, prevented or reduced respectively Roscovitine LTP induction, demonstrating that Ca^2+^ influx through L-type channels and NMDA receptors was important for triggering striatal LTP induced by Roscovitine. NMDA inhibition did not fully prevent LTP induction as previously reported (Calabresi et al., 1992b; Mahon et al., 2004; Lovinger, 2010). In those experiments where AP5 was present, the amplitude increase of the population spike persisted in a 26% compared to control. This result indicated that part of the increment in the synaptic amplitude was mediated by AMPA/KA receptor modulation as reported elsewhere (Svenningsson et al., 2004). As for L channels, its inhibition totally abolished LTP induction by Roscovitine. It has been reported that L-type Ca channels were involved in striatal LTD induction (Wang et al., 2006); however, there were no studies relating L-type Ca channels with corticostriatal LTP. Cdk5 may modulate L-type Ca channels directly or indirectly through PKA activation; if the PKA pathway remains active due to the inhibition of Cdk5, PKA may modulate other cellular substrates than DARPP-32 that participate in corticostriatal plasticity too. PKA activity induced by D1 receptor stimulation phosphorylates L-type Ca channels (Hernández-López et al., 1997) and NMDA receptors (Flores-Hernández et al., 2002), both increases Ca influx. Then during Roscovitine LTP induction, an important Ca^2+^ influx is mediated through NMDA receptors and L-type voltage-gated Ca^2+^ channels as reported previously (Kapur et al., 1998; Wankerl et al., 2010), favoring neuronal excitability (Mahon et al., 2004).

### Both types of MSN exhibited LTP in the presence of Roscovitine

Some MSNs express D1/SP (substance P) (direct pathway) receptors and other D2/ENK (Enkephalins) (indirect pathway) receptors, and it has been stated that MSNs from direct pathways express LTP through PKA signaling (Reynolds and Wickens, 2002), whereas the indirect pathway exhibits LTD through the stimulation of *endocannabinoid* receptors at the presynaptic cell (Lovinger, 2010). Nevertheless, cells from the indirect pathway can generate LTP through the stimulation of adenosine A2a receptors. A2a receptors are coupled to the cAMP/PKA pathway (Shen et al., 2008) and both neuronal types (direct and indirect pathways) are rich in DARPP-32 content (Bertran-Gonzalez et al., 2010). Our results in whole cell with D1 BAC mice showed that both types of neurons were able to express LTD in control and LTP in the presence of the Cdk5 inhibitor.

### Conclusion

Striatal LTD is induced by postsynaptic mechanisms; nevertheless, its expression is maintained through presynaptic mechanisms which reduce neurotransmitter release (Wang et al., 2006; Adermark et al., 2009; Lovinger, 2010). Cdk5 is widely expressed in pyramidal cells, which project into the striatum, from our results, we propose that Cdk5 inhibit neurotransmitter release in corticostriatal terminals and impede LTP expression. That is why inhibition of Cdk5 only at the postsynaptic cell did not affect LTD; it is the presynaptic Cdk5 activity, which favors LTD. For the modulation of LTP, Cdk5 inhibition has its effect at the postsynaptic level, modulating the cAMP/PKA/DARPP-32 pathway, which at the end may phosphorylate glutamate receptors (Roche et al., 1996) to increase the amplitude of the population spikes. These data together showed that Cdk5 inhibition induces LTP by decreasing LTD at the presynapse and favors LTP at the postsynapse.
